# Wind Turbines and Human Health

**DOI:** 10.3389/fpubh.2014.00063

**Published:** 2014-06-19

**Authors:** Loren D. Knopper, Christopher A. Ollson, Lindsay C. McCallum, Melissa L. Whitfield Aslund, Robert G. Berger, Kathleen Souweine, Mary McDaniel

**Affiliations:** ^1^Intrinsik Environmental Sciences Inc., Mississauga, ON, Canada; ^2^Intrinsik Environmental Sciences Inc., Venice, CA, USA

**Keywords:** wind turbines, human health, noise, electromagnetic fields, annoyance, infrasound, low-frequency noise, shadow flicker

## Abstract

The association between wind turbines and health effects is highly debated. Some argue that reported health effects are related to wind turbine operation [electromagnetic fields (EMF), shadow flicker, audible noise, low-frequency noise, infrasound]. Others suggest that when turbines are sited correctly, effects are more likely attributable to a number of subjective variables that result in an annoyed/stressed state. In this review, we provide a bibliographic-like summary and analysis of the science around this issue specifically in terms of noise (including audible, low-frequency noise, and infrasound), EMF, and shadow flicker. Now there are roughly 60 scientific peer-reviewed articles on this issue. The available scientific evidence suggests that EMF, shadow flicker, low-frequency noise, and infrasound from wind turbines are not likely to affect human health; some studies have found that audible noise from wind turbines can be annoying to some. Annoyance may be associated with some self-reported health effects (e.g., sleep disturbance) especially at sound pressure levels >40 dB(A). Because environmental noise above certain levels is a recognized factor in a number of health issues, siting restrictions have been implemented in many jurisdictions to limit noise exposure. These setbacks should help alleviate annoyance from noise. Subjective variables (attitudes and expectations) are also linked to annoyance and have the potential to facilitate other health complaints via the nocebo effect. Therefore, it is possible that a segment of the population may remain annoyed (or report other health impacts) even when noise limits are enforced. Based on the findings and scientific merit of the available studies, the weight of evidence suggests that when sited properly, wind turbines are not related to adverse health. Stemming from this review, we provide a number of recommended best practices for wind turbine development in the context of human health.

## Introduction

Wind power has been harnessed as a source of energy around the world for decades. Reliance on this form of energy is increasing. In 1996, the global cumulative installed wind power capacity was 6,100 MW; in 2011, that value had grown to 238,126 MW and at the end of 2013 it was 318,137 MW (1). While public attitude is generally overwhelmingly in favor of wind energy, this support does not always translate into local acceptance of projects by all involved (2). Opposition groups point to a number of issues concerning wind turbines, and possible effects on human health is one of the most commonly discussed. Indeed, a small proportion of people that live near wind turbines have reported adverse health effects such as (but not limited to) ringing in ears, headaches, lack of concentration, vertigo, and sleep disruption that they attribute to the wind turbines. This collection of effects has received the colloquial name “Wind Turbine Syndrome” (3).

The reason for the self-reported health effects is highly debated and information fueling this debate is found primarily in four sources: peer-reviewed studies published in scientific journals, government agency reports, legal proceedings, and the popular literature and internet. Some argue that reported health effects are related wind turbine operational effects [e.g., electromagnetic fields (EMF), shadow flicker from rotor blades, audible noise, low-frequency noise (LFN) and infrasound]; others suggest that when turbines are sited correctly, reported effects are more likely attributable to a number of subjective variables, including nocebo responses, where the etiology of the self-reported effect is in beliefs and expectations rather than a physiologically harmful entity (4–8). In 2011, Knopper and Ollson (9) published a review that contrasted the human health effects that had been purported to be caused by wind turbines in popular literature sources with what had been reported in the peer-reviewed scientific literature as well as by various government agencies. At that time, only 15 articles in the peer-reviewed scientific literature that specifically addressed issues related to human health and wind turbines were available [i.e., (4, 5, 10–22)].

Based on their review, Knopper and Ollson (9) concluded that although there was evidence to suggest that wind turbines can be a source of annoyance to some people, there was no evidence demonstrating a direct causal link between living in proximity to wind turbines and more serious physiological health effects. Furthermore, although annoyance has been statistically significantly associated with wind turbine noise [especially at sound pressure levels >40 dB(A)], a convincing body of evidence exists to show that annoyance is more strongly related to visual cues and attitude than to wind turbine noise itself. In particular, this was highlighted by the fact that people who benefit economically from wind turbines (e.g., those who have leased their property to wind farm developers) reported significantly lower levels of annoyance than those who received no economic benefit, despite increased proximity to the turbines and exposure to similar (or louder) sound levels.

In the years following the publication of Knopper and Ollson (9), the debate surrounding the relationship between wind turbines and human health has continued, both in the public and within the scientific community. In this review, we provide a bibliographic-like summary and analysis of the science around this issue specifically in terms of noise (including audible, LFN, and infrasound), EMF, and shadow flicker. Stemming from this review, we provide weight of evidence conclusions and a number of best practices for wind turbine development in the context of human health.

## Methods

The authors worked with a professional Health Sciences Information Specialist to develop a search strategy of the literature. Combinations of key words (i.e., annoyance, noise, environmental change, sleep disturbance, epilepsy, stress, health effect(s), wind farm(s), infrasound, wind turbines(s), LFN, EMF, wind turbine syndrome, neighborhood change) were entered into PubMed, the Thomson Reuters Web of Knowledge^SM^ and Google. No date restrictions were entered and literature was assessed up to the submission date of this manuscript (April 2014). The review was conducted in the spirit of the evaluation process outlined in the Cochrane Handbook for Systematic Reviews of Interventions.

As of the publication date of this review, there are close to 60 scientific peer-reviewed articles on the topic. Sources of information other than peer-reviewed scientific literature (e.g., websites, opinion pieces, conference proceedings, unpublished documents) were purposely excluded in this review because they are often unreliable and provide information that is typically anecdotal in nature or not traceable to scientific sources. A general summary, and key words of the articles reviewed herein, are presented in Table 1. These summaries provide results as they were reported by the authors of the articles and are without secondary interpretation.

**Table 1:** **General summary of reviewed articles**.

General topic	Authors	Source	Key words	General summary
Audible noise	Shepherd et al. (23)	Noise and Health	Health-related quality of life (HRQOL)	Cross-sectional study involving questionnaires about quality of life living near and away from turbines. Statistically significant differences were noted in some HRQOL scores; residents within 2 km of a turbine reporting lower overall quality of life, physical quality of life, and environmental quality of life
	Janssen et al. (24)	Journal of the Acoustical Society of America	Annoyance, economic benefit, sensitivity, visual cues	Expanded on the datasets collected by Pedersen and Persson Waye (4, 5) and Pedersen et al. (17) in Sweden and the Netherlands. Authors evaluated self-reported annoyance indoors and outdoors compared to sound levels (Lden) from wind turbines. Like the authors before them who relied on these datasets, found that annoyance decreased with economic benefit and may have increased with noise sensitivity, visibility, and age. In comparison to other sources of environmental noise, annoyance due to wind turbine noise was found at relatively low noise exposure levels
	Verheijen et al. (25)	Science of the Total Environment	Annoyance, noise limits	Objective was to assess proposed Dutch standards for wind turbine noise and consequences for people and feasibility of meeting energy policy targets. Authors used a combination of audible and low-frequency noise models and functions to predict existing level of severely annoyed people living around existing wind turbines in the Netherlands. Found that at 45 dB(Lden) severe annoyance due to low-frequency noise unlikely; suggested that this noise limit is suitable as a trade-off between the need for protection against noise annoyance and the feasibility of national targets for renewable energy
	Bakker et al. (26)	Science of the Total Environment	Annoyance, distress, economic benefit, sleep disturbance	A dose–response relationship was found between immission levels of wind turbine sound and self-reported noise annoyance. Sound exposure was also related to sleep disturbance and psychological distress among those who reported that they could hear the sound, however not directly but with noise annoyance. Respondents living in areas with other background sounds were less affected than respondents in quiet areas. Found that people, animals, traffic and mechanical sounds were more often identified as a source of sleep disturbance than wind turbines
	Nissenbaum et al. (27)	Noise and Health	Epworth Sleepiness Score (ESS), Pittsburgh Sleep Quality Index (PSQI), SF36v2	Purpose of the investigations was to determine the relationship between reported adverse health effects and wind turbines among residents of two rural communities. Participants living 375–1,400 m and 3.3–6.6 km were given questionnaires to obtain data about sleep quality, daytime sleepiness and general physical and mental health. Authors reported that when compared to people living further away than 1.4 km from wind turbines, those people living within 1.4 km of wind turbines had worse sleep, were sleepier during the day and had worse mental health scores
	Ollson et al. (28)	Noise and Health	Rebuttal to Nissenbaum et al. (27)	Suggested that Nissenbaum et al. (27) extended their conclusions and discussion beyond the statistical findings of their study and that they did not demonstrated a statistical link between wind turbines – distance – sleep quality – sleepiness and health. In fact, their own statistical findings suggest that although, scores may be statistically different between near and far groups for sleep quality and sleepiness, they are no different than those reported in the general population. The claims of causation by the authors (i.e., wind turbine noise) for negative scores are not supported by their data
	Barnard (29)	Noise and Health	Rebuttal to Nissenbaum et al. (27)	Pointed out a number of problems with Nissenbaum et al. (27) study and suggested that data presented do not justify the very strong conclusions reached by the authors
Audible noise (continued)	Mroczek et al. (30)	Annals of Agricultural and Environmental Medicine	SF-36, Visual Analog Scale (VAS)	Purpose of study was to assess how people’s quality of life is affected by the close proximity of wind farms. Authors found that close proximity of wind farms does not result in the worsening of the quality of life based on the Norwegian version of the SF-36 General Health Questionnaire, the Visual Analog Scale (VAS) for health assessment, and original questions
	Taylor et al. (31)	Personality and Individual Differences	Personality traits	Study examined the influence of negative oriented personality (NOP) traits on the effects of wind turbine noise and reporting on non-specific symptoms (NSS). Results of the study showed that while calculated actual wind turbine noise did not predict reported symptoms, perceived noise did
	Evans and Cooper (32)	Acoustics Australia	Predicted and measured noise levels	A comparison of predicted noise levels from four commonly applied prediction methods against measured noise levels from six operational wind farms (at 13 locations) in accordance with the applicable guidelines in South Australia. Results indicate that the methods typically over-predicted wind farm noise levels but that the degree of conservatism appeared to depend on the topography between the wind turbines and the measurement location
	Maffei et al. (33)	International Journal of Environmental Research and Public Health	Visual cues, perception	Investigated the effects of the visual impact of wind turbines on the perception of noise. Found distance was a strong predictor of an individual’s reaction to the wind farm; data showed that increased distance resulted in a more positive general evaluation of the scenario and decreased perceived loudness, noise annoyance, and stress caused by sound. Found the color of the wind turbines (base and blade stripes) impacted an individuals’ perception of noise
	Van Renterghem et al. (34)	Science of the Total Environment	Annoyance, attitude, laboratory experiment, visual cues	Conducted a two-stage listening experiment to assess annoyance, recognition, and detection of noise from a single wind turbine. Results support the hypothesis that non-noise variables, such as attitude and visual cues, likely contributed to the observation that people living near wind turbines (who do not receive an economic benefit from the turbines) report higher levels of annoyance at lower sound pressure levels than would be predicted for other community noise sources
	Baxter et al. (35)	Energy Policy	Risk perception, economic benefit, community conflict, policy	Conducted a study to investigate the role of health risk perception, economic benefit, and community conflict on wind turbine policy. Two communities were assessed: one located in proximity to two operating wind farms and a control community without turbines. Authors found that residents from the community with operational wind energy projects were more supportive of wind turbines than residents in the area without turbines
	Chapman et al. (6)	PLoS One	Psychogenic effects, nocebo, community complaints	Provided an overview of the growing body of literature supporting the notion that the attribution of symptoms and disease to wind turbine exposure is a modern health worry. Suggested that nocebo effects likely play an important role in the observed increase in wind farm-related health complaints. Suggested that reported historical and geographical variations in complaints were consistent with “communicated diseases” with nocebo effects likely to play an important role in the etiology of complaints rather than direct effects from turbines
	Whitfield Aslund et al. (36)	Energy Policy	Predicted annoyance, modeling	Used previously reported dose–response relationships between wind turbine noise and annoyance to predict the level of community noise annoyance that may occur in the province of Ontario. The results of this analysis indicate that the current wind turbine noise restrictions in Ontario will limit community exposure to wind turbine related noise such that levels of annoyance are unlikely to exceed previously established background levels of noise-related annoyance from other common noise sources
Low-frequency noise and infrasound	Møller and Pedersen (37)	Journal of the Acoustical Society of America	Annoyance, insulation, indoor sound levels	Conducted a low-frequency noise study from four large turbines (>2 MW) and 44 other small and large turbines (7 > 2 MW and 37 < 2 MW). Low-frequency sound insulation was measured for 10 rooms under normal living conditions in houses exposed to low-frequency noise. Concluded that the spectrum of wind turbine noise moves down in frequency with increasing turbine size. Suggested that the low-frequency part of the noise spectrum plays an important role in the noise at neighboring properties. They hypothesized that if the noise from the investigated large turbines had an outdoor level of 44 dB(A) there was a risk that a substantial proportion of the residents would be annoyed by low-frequency noise, even indoors
	Bolin et al. (38)	Environmental Research Letters	Health effects, review, turbulence	Conducted a literature review over a 6-month period ending April 2011 into the potential health effects related to infrasound and low-frequency noise exposure surrounding wind turbines. Concluded that empirical support was lacking for claims that low-frequency noise and infrasound cause serious health affects in the form of “vibroacoustic disease,” “wind turbine syndrome,” or harmful effects on the inner ear
	Rand et al. (39)	Bulletin of Science, Technology and Society	Indoor sound levels, health effects, acute effects	Studies took place over a 2-day period inside a home where people were self-reporting serious adverse health effects. Authors reported on wind speed at hub of turbine, dB(A) and dB(G) filtering indoors and outdoors. Reported on acute effects
	Ambrose et al. (40)	Bulletin of Science, Technology and Society	
	Turnbull et al. (41)	Acoustics Australia	Underground measurement, comparative study	Developed an underground technique to measure infrasound. Measured infrasound at two Australian wind farms as well as in the vicinities of a beach, a coastal cliff, the city of Adelaide, and a power station. Reported that the measured levels at wind farms below the audibility threshold and similar to that of urban and coastal environments and near other engineered noise sources. Level of infrasound from wind farms at 360 and 85 m [61 and 72 dB(G), respectively] was comparable to that observed at a distance of 25 m from ocean waves [75 dB(G)]
	Crichton et al. (7)	Health Psychology	Negative expectations, symptom reporting, laboratory experiment	Examined the possibility that expectations of negative health effects from exposure to infrasound promote symptom reporting using a sham controlled, double-blind provocation study. Participants in the high-expectancy group reported significant increases in the number and intensity of symptoms experienced during exposure to both infrasound and sham infrasound. Conversely, there were no symptomatic changes in the low-expectancy group
	Crichton et al. (8)	Health Psychology	Negative and positive expectations, symptom reporting, laboratory experiment	Authors investigated how positive expectations can produce a reduction in symptoms. Expectations were found to significantly alter symptom reporting: participants who were primed with negative expectations became more symptomatic over time, suggesting that their experiences during the first exposure session reinforced expectations and led to heightened symptomatic experiences in subsequent sessions
Electromagnetic fields	Havas and Colling (42)	Bulletin of Science, Technology and Society	Poor power quality, ground current, electrical hypersensitivity	Authors hypothesized that symptoms of some living near wind turbines could be caused by electromagnetic waves in the form of poor power quality (dirty electricity) and ground current resulting in health effects in those that are electrically hypersensitive. Indicated that individuals reacted differently to both sound and electromagnetic waves and this could explain why not everyone experienced the same health effects living near turbines
	Israel et al. (43)	Environ-mentalist	Vibration measurement, noise, risk	Conducted EMF, sound, and vibration measurements at wind energy parks in Bulgaria. Concluded that EMF levels were not of concern from wind farm
	McCallum et al. (44)	Environ-mental Health	Variable distances and wind, residential measures	Magnetic field measurements were collected in the proximity of 15 wind turbines, two substations, buried and overhead collector and transmission lines and nearby homes. Results suggest there is nothing unique to wind farms with respect to EMF exposure; in fact, magnetic field levels in the vicinity of wind turbines were lower than those produced by many common household electrical devices and were well below any existing regulatory guidelines with respect to human health
Review articles, editorials and social commentaries	Bulletin of Science, Technology and Society (BSTS) Special Edition	Bulletin of Science, Technology and Society	Various authors, health effects, social commentary, opinion pieces	Special edition made up of nine articles devoted entirely to wind farms and potential health effects. Many of the articles in the special edition were written as opinion pieces or social commentaries
	Hanning and Evans (45)	British Medical Journal	Sleep disturbance	Purpose was to opine on the relationship between wind turbines noise and health effects. Suggested that a large body of evidence exists to suggest that wind turbines disturb sleep and impair health at distances and external noise levels that are permitted in most jurisdictions
	Chapman (46)	British Medical Journal	Weight of evidence	In a rebuttal to Hanning and Evans (45) Chapman points to 17 independent reviews of the literature around wind turbines and human health that contrast the opinion of Hanning and Evans
	Farboud et al. (47)	Journal of Laryngology and Otology	Low-frequency noise (LFN), infrasound (IS), inner ear physiology, wind turbine syndrome	Conducted a literature search for articles published within the last 10 years, using the PubMed database and the Google Scholar search engine, to look at the effects of low-frequency noise and infrasound. Suggested the evidence available was incomplete and until the physiological effects of LFN and infrasound were fully understood, it was not possible to conclusively state that wind turbines were not causing any of the reported effects
	McCubbin and Sovacool (48)	Energy Policy	Comparative study, natural gas, health, and environmental benefits	Compared the health and environmental benefits of wind power in contrast to natural gas
	Roberts and Roberts (49)	Journal of Environmental Sciences	PubMed-based review, low-frequency noise (LFN), infrasound (IS), health effects	Conducted a summary of the peer-reviewed literature on the research that examined the relationship between human health effects and exposure to low-frequency sound and sound generated from the operation of wind turbines. Concluded that a specific health condition or collection of symptoms has not been documented in the peer-reviewed, published literature that has been classified as a “disease” caused by exposure to sound levels and frequencies generated by the operations of wind turbines
Review articles, editorials and social commentaries (continued)	Chapman and St. George (50)	Australian and New Zealand Journal of Public Health	Vibroacoustic disease (VAD); factoid	Investigated the extent to which VAD and its alleged association with wind turbine exposure had received scientific attention, the quality of that association and how the alleged association gained support by wind farms opponent. Based on a structured scientific database and Google search strategy, the authors showed that VAD has received virtually no scientific recognition and that there is no evidence of even rudimentary quality that vibroacoustic disease is associated with or caused by wind turbines. Stated that an implication of this “factoid” – defined as questionable or spurious statements – may have been contributing to nocebo effects among those living near turbines
	Jeffery et al. (51)	Canadian Family Physician	Health effects	Overall goal of these commentary pieces was to provide information to physicians regarding the possible health effects of exposure to noise produced by wind turbines and how these may manifest in patients
	Jeffery et al. (52)	Canadian Journal of Rural Medicine		

Through the systematic review process, it was evident that there was significant variability in both the measures of exposure (i.e., proximity to turbines, field noise measures, lab noise measures, or magnetic field measurements) and the health outcomes examined (i.e., annoyance, sleep scores, and various quality of life metrics). The methodological heterogeneity in study designs across the selected health-based investigations inhibited a quantitative combination of results. In other words, meta-analytic methods were not appropriate for this updated systematic review of the literature on wind turbine and health effect. Rather qualitative interpretation is provided.

## Results

### Overall noise

Knopper and Ollson (9) reviewed a number of studies that examined the noise levels produced by wind turbines, perception of wind turbine noise, and/or responses to wind turbine noise [e.g., (4, 5, 10, 12, 13, 15–18, 21)]. The results of more recent studies that investigated wind turbine noise with respect to potential human health effects are summarized below in chronological order of publication.

Shepherd et al. (23): Shepherd et al. reported on a cross-sectional study comparing health-related quality of life (HRQOL) of people living in proximity (i.e., <2 km) to a wind farm to a control group living >8 km away from the nearest wind farm. It involved self-administered questionnaires that included the World Health Organization (WHO) quality of life scale, in semi-rural New Zealand. The turbine group was drawn from residents of 56 homes in South Makara Valley, all within 2 km of a wind turbine. General outdoor noise levels in the area, obtained from a conference proceeding by Botha (53), were reported to range from 24 to 54 dB(A). The comparison group was taken from 250 homes in a geographically and socioeconomically matched area, at least 8 km from any wind farm in the region. General outdoor noise levels for the comparison group were not reported. The questionnaire was named the “*2010 Well-being and Neighborhood Survey*” in order to mask the true intent of the study and reduce bias against wind turbines. This is similar to the work of Pedersen in Europe, in that the surveys were not explicitly about wind turbines. Response rates were 34% from the Turbine group (number of participants *n* = 39) and 32% from the Comparison group (*n* = 158).

Overall, Shepherd et al. reported statistically worse (*p* < 0.05) scores in the Turbine group for physical HRQOL, environmental QOL and HRQOL in general. There was no statistical difference in social or psychological scores. Based on these results, the authors concluded that “utility-scale” wind energy generation was not without adverse health impacts on nearby residents and suggested setback distances need to be >2 km in hilly terrain. However, there are a number of limitations in this study that undermine the conclusion stated above. One key concern is that the results were based on only a limited number of participants (*n* = 39) for the Turbine group. In comparison, the survey datasets compiled in Sweden and the Netherlands by Pedersen and Persson Waye (4, 5) and Pedersen et al. (17), respectively, involved a total of 1,755 respondents overall. In these surveys, the only response found to be significantly related to A-weighted wind turbine noise exposure was annoyance, even though a number of physiological and psychological variables were also investigated. In addition, Shepherd et al. did not discuss the impact of participants’ attitudes or visual cues that may have influenced the reports of decreased HRQOL. Given that other studies have indicated that annoyance was more closely related to visual cues and attitude, this could provide further explanation of why overall HRQOL scores were lower in the Turbine group. Presumably all residents within 2 km of a turbine would be able to see one, or more, of the turbines. Furthermore, although it was implied in the title of the article that noise from wind turbines was causing the observed effects, the study did not include either measured or estimated wind turbine noise exposure values for the individual survey respondents. Therefore, they were unable to demonstrate a dose–response relationship between the observed responses and exposure to wind turbine noise. In light of this, as recognized by Shepherd et al. (23), it is possible that the observed effects were driven by other causes such as conflicts between the community and the wind farm developers rather than a direct result of noise exposure. Based on the limitations discussed above, we consider that the authors’ recommendation for a 2 km setback distance was not supported by the evidence presented in this study.

Janssen et al. (24): expanding on the datasets collected by Pedersen and Persson Waye (4, 5) and Pedersen et al. (17) in Sweden and the Netherlands, Janssen et al. evaluated self-reported annoyance indoors and outdoors compared to sound levels (Lden) from wind turbines. To derive the Lden, the authors added a correction factor of 4.7 dB(A) to outdoor A-weighted sound pressure levels from the datasets used in the previous studies. Annoyance in this study was ranked on a 4-point scale: 1 was “not annoyed,” 2 was “slightly annoyed,” 3 was “rather annoyed,” and 4 was “very annoyed.” Visual cue (“Can you see a wind turbine from your dwelling or your garden/balcony?”), economic benefit [“Are you a (co)owner of one or more wind turbines?”], and noise sensitivity (on either a 4 or 5 point scale with 1 representing “not sensitive” and 4 or 5 representing “very/extremely sensitive”) were also assessed. Like the authors before them who relied on these datasets, Janssen et al. found that annoyance decreased with economic benefit and may have increased with noise sensitivity, visibility, and age. Rates of annoyance indoors from wind turbines to industrial noise from stationary sources and air, road and rail noise were also compared and it was concluded that: “…*annoyance due to wind turbine noise is found at relatively low noise exposure levels” and that “some similarity is found in the range Lden 40–45 dB between the percentage of annoyed persons by wind turbine noise and aircraft noise.”*

Verheijen et al. (25): the objective of this study was to assess the proposed Dutch protective standards for wind turbine noise, both on consequences for inhabitants and feasibility of meeting energy policy targets. The authors used a combination of audible and LFN models and functions derived by Janssen et al. (24) to predict the existing level of severely annoyed people living around existing wind turbines in the Netherlands. They estimated that there were approximately 1,500 severely annoyed individuals, in a total population of approximately 440,000 living at sound levels of 29 dB(Lden) around wind turbines. The authors reported that: *“For The Netherlands, a socially acceptable percentage of severely annoyed lies around 10%, which can be derived from the existing limits and dose–response functions of railway and road noise. This would result in an acceptable noise reception limit for wind turbines of about 47 to 49 dB.”* The authors decided to examine the feasibility of lowering the limit below 47–49 dB(Lden). They estimated that it may be feasible from a land mass perspective to lower the noise limit to 40 dB(Lden); however, given that lands are often rejected due to reasons other than noise that another value should be selected. They stated *“The percentage of severely annoyed at 45 dB is rated at 5.2% for wind turbine noise, which is well below 10% that corresponds to the existing road and railway traffic noise limits.”* They also determined that, at 45 dB(Lden), severe annoyance effects due to LFN were unlikely and suggested that this noise limit suited as a trade-off between the need for protection against noise annoyance and the feasibility of national targets for renewable energy.

Bakker et al. (26): the purpose of this study was to evaluate the relationship between exposure to the sound of wind turbines and annoyance, self-reported sleep disturbance, and psychological distress of people that live in their vicinity. This investigation relied on survey data, previously reported and discussed by Pedersen et al. (17), collected from 725 residents of the Netherlands living in the vicinity of wind turbines. As reported by Pedersen et al. (17), survey respondents answered questions about environmental factors and road traffic noise (and wind noise) as well as the effect of wind turbines on annoyance, sleep disturbance, and psychological distress.

Bakker et al. differed from Pedersen et al. (17) in that it provided a direct comparison of people who economically benefited from turbines with those who did not, specifically in relation to annoyance. Bakker et al. (26) reported that only 3% of survey respondents receiving economic benefit from wind turbines reported being “rather annoyed” or “very annoyed” by wind turbine noise when outdoors, while none reported being rather or very annoyed by wind turbine noise when indoors. In comparison, the proportions of survey respondents who did not receive an economic benefit who reported being rather or very annoyed indoors and outdoors were 12 and 8%, respectively, even though they were exposed to significantly lower levels of wind turbine sound.

What is more, Bakker et al. also compared sound-related sources of sleep disturbance in rural and urban areas in respondents who did not benefit economically from wind turbines. They found that people, animals, traffic, and mechanical sounds were more often identified as a source of sleep disturbance than wind turbines. In fact, in rural areas, only 6% of people identified wind turbines as the sound source of sleep disturbance compared to 11.7% for people/animals and 12.5% for traffic/mechanical sounds. In urban areas, only 3.8% of people identified wind turbines as the sound source of sleep disturbance compared to 14.4% for people/animals and 16.9% for traffic/mechanical sounds.

Nissenbaum et al. (27), Ollson et al. (28), and Barnard (29): the stated purpose of the investigations conducted by Nissenbaum et al. was to determine the relationship between reported adverse health effects and wind turbines among residents of two rural communities. Participants living 375–1,400 m and 3.3–6.6 km were given questionnaires to obtain data about sleep quality [using the Pittsburgh Sleep Quality Index (PSQI)], daytime sleepiness [using the Epworth Sleepiness Score (ESS)], and general physical and mental health (MH) (using the SF36v2 health survey). Overall, the authors reported that when compared to people living further away than 1.4 km from wind turbines, those people living within 1.4 km of wind turbines had worse sleep, were sleepier during the day, and had worse MH scores. Based on these findings the authors concluded that: *“*…*the noise emissions of IWTs disturbed the sleep and caused daytime sleepiness and impaired mental health in residents living within 1.4 km of the two IWT installations studied.”*

In a subsequent issue of Noise and Health, two letters to the editor were published that were critical of this study and its conclusions (28, 29). In particular, the letter from Barnard (29) criticized the statistical analysis in Nissenbaum et al. (27), which stated that there was a “strong” dose–response relationship between distance to the nearest wind turbine and both the “PSQI” and the “Epworth Sleepiness Scale.” Barnard stated: *“I cannot see how this is justified, given the presented data. In contrast to the conclusions, Figure 1 and Figure 2 in the paper*… *show a very weak dose-response, if there is one at all. The near horizontal ‘curve fits’ and large amount of ‘data scatter’ are indications of the weak relationship between sleep quality and turbine distance. The authors seem to use a low P value as a support for the hypothesis that sleep disturbance is related to turbine distance. A better interpretation of the P value related to a near horizontal line fit would be that it suggests a high probability of a weak-dose response. Correlation coefficients are not given, but should have been given, to indicate the quality of the curve fits.”* Ollson et al. (28) pointed out that Nissenbaum et al. extended their conclusions and discussion beyond the statistical findings of their study. They stated “*We believe that they have not demonstrated a statistical link between wind turbines – distance – sleep quality – sleepiness and health. In fact, their own statistical findings suggest that although, scores may be statistically different between near and far groups for sleep quality and sleepiness, they are not different than those reported in the general population. The claims of causation by the authors (i.e., wind turbine noise) for negative MCS scores are not supported by their data. This work is exploratory in nature and should not be used to set definitive setback guidelines for wind-turbine installations.”*

Mroczek et al. (30): Mroczek et al. published the results of a study conducted in 2010 that evaluated the impact of living in close proximity to wind turbines on an individual’s perceived quality of life. The study group consisted of 1,277 randomly selected Polish adults (703 women and 574 men) living in the vicinity of wind farms. The different distance (house to turbine) groups were: <700 m, from 700 to 1000 m, from 1,000 to 1,500 m, and >1,500 m. The quality of life was measured using the Norwegian version of the SF-36 General Health (GH) Questionnaire, the Visual Analog Scale (VAS) for health assessment, and some original questions about approximate distance to wind farm, age, gender, education, and profession. The SF-36 (Short Form 36) Questionnaire consists of 36 questions divided into 8 subscales: physical functioning (PF), role functioning physical (RP), bodily pain (BP), GH, vitality (V), social functioning (SF), role functioning emotional (RE), MH, and one additional question regarding health changes.

According to the authors “*The respondents assessed their health through answering questions included in the SF-36 and VAS. They were asked to mark the point corresponding with their well-being on the level from 0 to 100, where 0 denoted the worst possible state of health and 100 – excellent health*.” The results showed that regardless of the distance from the wind farm (i.e., from <700 to >1,500 m) respondents ranked their PF scores as highest out of all of the quality of life components. Overall, people living closest to wind farms assessed their quality of life as higher than those living in more distant areas. The scores for the MH component, GH, SF, and RE were highest in the group living closest to the wind farms and lowest by those living greater than 1.5 km away. The authors noted that there may have been confounding factors that contributed to the observed results (e.g., economic factors). Since other studies have shown links between self-reported health status, proximity to wind turbines and the direct influence of economic benefit on levels of annoyance [e.g., (17, 26)], these major confounding factors also need to be considered when interpreting the results of the Mroczek et al. study on quality of life and proximity to wind turbines.

Taylor et al. (31): this study examined the influence of negative oriented personality (NOP) traits on the effects of wind turbine noise and reporting on non-specific symptoms (NSS). The study was conducted based on the hypothesis that the public has become increasingly concerned with attributing NSS to environmental features (e.g., wind turbines). The study focused on three NOP traits in particular: neuroticism (N), negative affect (NA), and frustration intolerance (FI). The authors noted that previous research has demonstrated that individuals with high N and NA typically evaluate their environment more negatively. Furthermore, FI may have impacted the way an individual perceived and evaluated environmental factors from an inability to bear or cope with perceived negative emotions, thoughts and events. A survey was mailed out to 1,270 households within 500 m of eight 0.6 kW turbine installations and within 1 km of four 5 kW turbines in two cities in the U.K. Individuals within the household (>18 years old) could anonymously complete the survey and mail the results back or submit them online. In total, 138 completed surveys were returned. Actual sound levels were calculated for those households who completed the survey, and participants were asked to describe the perceived noise, including the type of noise (e.g., swooshing, whistling, buzzing), frequency, and loudness (based on a 0–4 ranking scale). Participants were also asked a series of questions to determine the level of NOP traits and related health/symptom reporting information.

The results of the study showed that while calculated actual wind turbine noise did not predict reported symptoms, perceived noise did. Specifically: “*…for those higher in NOP traits, there was a stronger link between perceived noise and symptom reporting. There was however, no relationship between calculated actual noise from the turbine and participants attitude to wind turbines. This means that those who had a more negative attitude to wind turbines perceived more noise from the turbine, but this effect was not simply due to individuals being able to actually hear the noise more*.”

Evans and Cooper (32): in their paper called “Comparison of predicted and measured wind farm noise levels and implications for assessments of new wind farms,” Evans and Cooper present a comparison of predicted noise levels from four commonly applied prediction methods against measured noise levels from six operational wind farms (conducted at 13 locations) in accordance with the applicable guidelines in South Australia. The results indicate that the methods typically over-predicted wind farm noise levels but that the degree of conservatism appeared to depend on the topography between the wind turbines and the measurement location. Briefly, Evans and Cooper found that the commonly used ISO 9613-2 model (with completely reflective ground) and the CONCAWE model generally over-predicted noise levels by 3–6 dB(A), but the amount of over-prediction was related to the topography (i.e., relatively flat topography or a steady slope from the turbines). However, at sites where there was a significant concave slope from the turbines down to the measurement sites, these commonly used prediction methods were typically accurate, with the potential of marginal under-prediction in some cases (when ISO 9613-2 used 50% absorptive ground).

A requirement of many regulatory agencies is that noise modeling be conducted by developers prior to the construction of wind turbines. A common criticism of this approach is that modeled values are not representative of actual noise from operational wind farms. Evans and Cooper’s findings show that this is not the case, but caution about the role of topography.

Maffei et al. (33): despite the fact that wind farms are represented as environmentally friendly projects, wind turbines are viewed by some as visual and audible intruders that spoil the landscape and generate noise. Consequently, Maffei et al. (33) conducted a study investigating the effects of the visual impact of wind turbines on the perception of noise. The study consisted of 64 participants (34 males, 30 females) who resided in either urban or rural areas. Participants were asked to fill out a questionnaire to obtain information regarding age, gender, education, and local neighborhood characteristics. A number of statements were then submitted to the participants where they were asked to respond based on a 100-point Likert scale ranging from “disagree strongly” to “agree strongly.” The statements were based on personal views about green energy, wind turbines, noise, and other related subject matter. Subsequently, a virtual reality scenario was created to emulate the visual impact of a wind farm on a rural landscape and included an audio component recorded from a 16 turbine wind farm in Frigento, Italy. In total, three factors were manipulated in the experiment: distance from the wind farm (150, 250, and 500 m); the number of wind turbines (1, 3, and 6); the color of the base of the turbine and any stripes on the blades (white, red, brown, green). Each participant was asked to view all of the scenarios using a 3D visor and asked to respond to a number of questions pertaining to perceived loudness, sound pleasantness, noise annoyance, sound stress, sound tranquility, and visual pleasantness.

The results found that distance was a strong predictor of an individual’s reaction to the wind farm. In particular, the data showed that increased distance resulted in a more positive general evaluation of the scenario and decreased perceived loudness, noise annoyance, and stress caused by sound. Additionally, the authors found that the color of the wind turbines (base and blade stripes) impacted an individuals’ perception of noise. Generally, white and green turbines were preferred to brown and red ones. Specifically, green turbines scored the highest since they were perceived as being the “most integrated” into the landscape. The authors concluded that their results confirmed the interconnectedness between auditory and visual components of individual perception.

Van Renterghem et al. (34): Van Renterghem et al. (34) conducted a two-stage listening experiment to assess annoyance, recognition, and detection of noise from a single wind turbine. A total of 50 participants with “normal” hearing abilities participated in the experiment and were classified as having a positive to neutral attitude toward renewable energy. *In situ* recordings made at close distance (30 m downwind) from a 1.8 MW turbine operating at 22 rotations per minute (rpm) were mixed with road traffic noise and processed to simulate indoor sound pressure levels at 40 dB(LAeq). In the first stage, where participants were unaware of the true purpose of the experiment, samples were played during a quiet leisure activity. Under these conditions (i.e., when people were unaware of the different sources of noise), pure wind turbine noise produced similar annoyance ratings as unmixed highway noise at the same equivalent level, while annoyance from local road traffic was significantly higher. These results supported the hypothesis that non-noise variables, such as attitude and visual cues, likely contributed significantly to the observation that people living near wind turbines (who do not receive an economic benefit from the turbines) report higher levels of annoyance at lower sound pressure levels than would be predicted for other community noise sources [e.g., (17, 24)].

In the second stage of the Van Renterghem et al. (34) study, participants were allowed to listen to a recording of unmixed wind turbine sound [at 40 dB(A)] for 30 s in order to familiarize themselves with the sound. After this, they listened to 10 sets of paired sound samples; one of which contained unmixed road traffic noise and the other that contained wind turbine noise mixed with road traffic at signal-to-noise ratios varying between −30 dB(A) and +10 dB(A). For each pair, participants were asked to identify which of the two samples contained the wind turbine noise. The detection of wind turbine noise in the presence of highway noise was found a “signal-to-noise” ratio as low as −23 dB(A). This demonstrated that once the subject was familiar with wind turbine noise, it could easily be detected even in the presence of highway traffic noise. This could also help explain the increased rates of noise annoyance at home reported by Pedersen et al. (17) and Janssen et al. (24) since residents would be familiar with the sound and be able to discern it if they listened for it when primed by visual cues. Overall, the findings support the idea that noticing the sound could be an important aspect of wind turbine noise annoyance. Awareness of the source and recognition of the wind turbine sound was also linked to higher levels of annoyance. Van Renterghem et al. noted that: *“The experiment reported in this paper supports the hypothesis that previous observations, reporting that retrospective annoyance for wind turbine noise is higher than that for highway noise at the same equivalent noise level, is grounded in higher level appraisal, emotional, and/or cognitive processes. In particular, it was observed that wind turbine noise is not so different from traffic noise when it is not known beforehand.”*

Baxter et al. (35): in 2010, Baxter and colleagues conducted a study to investigate the role of health risk perception, economic benefit, and community conflict on wind turbine policy. The study, published in 2013, had two parts: a literature review and quantitative survey meant to determine perceptions of wind turbines and how they are linked to support or opposition to wind turbines in the community. Two communities were assessed: one located in proximity to two operating wind farms and a control community without turbines. Overall, the authors found that residents from the community with operational wind energy projects (which were introduced prior to the *Green Energy Act* in Ontario) were more supportive of wind turbines than residents in the area without turbines (78 vs. 29%, with “support” defined as agreeing to vote in favor of local turbines). The authors also reported that residents in the turbine community were more accepting of turbine esthetics than people in the control community and less worried about health impacts, this despite the fact that the wind farms in the “case” group were in some cases closer to homes than currently permitted.

Baxter et al. indicated that the lack of support in the control community could have been due to political lobbying during the provincial election, where one candidate suggested a moratorium on wind turbine as part of their campaign. The authors also highlighted the role of health risk perception (which seemed linked to political lobbying) as a variable leading to the lack of support. The finding that “*Our study highlights the need to add health risk perception to the agenda for social research on turbines*” is valid, albeit dated in the Ontario context, since an integral part of any wind development project in Ontario is public consultation with wind turbines and health as a fundamental component. These findings supported the idea that perception of health risks is heavily impacted by expectation, media coverage, and that “hands on experience” could serve to increase familiarity and decrease concerns.

Chapman et al. (6): the authors provided an overview of the growing body of literature supporting the notion that the attribution of symptoms and disease to wind turbine exposure is a modern health worry. Chapman et al. also suggested that nocebo effects likely play an important role in the observed increase in wind farm-related health complaints. By evaluating records of complaints from wind farm companies about noise or health from residents living near 51 wind farms across Australia, two theories about the etiology of complaints were tested: one being direct effects from turbines and the other being “psychogenic” effects brought on by nocebo effects.

Chapman et al. found a number of historical and geographical variations in wind farm complaints from Australians.

1. Nearly 65% of Australian wind farms, 53% of which have turbines >1 MW, have never been subject to noise or health complaints. These farms have an estimated 21,633 residents within 5 km and have operated complaint-free for a cumulative 267 years. No complaints were reported in Western Australia and Tasmania.
2. One in 254 residents across Australia appeared to have ever complained about health and noise, and 73% of these residents live near 6 wind farms that have been targeted by anti-wind farm groups. Ninety percentage of complaints were made after anti-wind farm groups added health concerns to their wider opposition in 2009.
3. In the years after, health or noise complaints were rare despite large and small-turbine wind farms having operated for many years.

It was suggested that reported historical and geographical variations in complaints were consistent with “communicated diseases” with nocebo effects likely to play an important role in the etiology of complaints rather than direct effects from turbines. This novel work highlighted the role of negative expectations and how they could lead to the development of complaints near wind farms. These findings were supported by many other studies that were suggestive of subjective variables, rather than wind turbine specific variables, as the source of annoyance for some people.

Whitfield Aslund et al. (36): Whitfield Aslund et al. used previously reported dose–response relationships between wind turbine noise and annoyance to predict the level of community noise annoyance that may occur in the province of Ontario. Prediction for future wind farm developments (planned, approved, or in process) were compared to previously reported rates of annoyance that were associated with more common noise sources (e.g., road traffic). Modeled noise levels and distance to the nearest wind farm-related noise source were compiled for over 8,000 individual receptor locations (i.e., buildings, dwellings, campsites, places of worship, institutions, and/or vacant lots) from 13 wind power projects in the province of Ontario that had been approved since 2009 or were under Ministry of the Environment (MOE) review as of July 2012. This information was then compared to the wind turbine noise specific dose–response relationships for self-reported annoyance from Pedersen et al. (17) and Bakker et al. (26) using data collected from 725 survey respondents living in the proximity of wind turbines (<2.5 km) in the Netherlands.

One of the study findings was that a distinct exponentially decreasing relationship was observed between distance to the nearest noise source and the sound pressure level predicted. However, although distance to the nearest noise source could explain a large proportion (86%) of the total variance in predicted sound pressure levels, other sources of variation are also important; predicted sound pressure levels at a set distance varied by approximately 5–10 dB(A) and the distance at which a set sound pressure level was met varied by approximately 1000 m. These variations reflect differences in the noise model inputs such as the physical design and noise emission ratings of the turbines (and transformer substations, if present) used in different projects and the total number of turbines (and transformer substations, if present) in the vicinity of the receptor location. Given that noise levels can vary substantially at a given distance, these data highlighted the inadequacy of using distance to the nearest turbine as a proxy for wind turbine noise exposure.

One of the other findings was that, for non-participating receptors, predicted rates of noise-related annoyance (when indoors) would not exceed 8%, with further reductions in the rates of annoyance at increased distances (i.e., >1 km). In comparison, it had previously been established that approximately 8% of adult Canadians reported being either “very or extremely bothered, disturbed, or annoyed” by noise in general when they were at home and 6.7% of adult Canadians indicated they were either “very or extremely annoyed” by traffic noise specifically (54). Even in small Canadian communities (i.e., <5000 residents) that are typically associated with low background noise levels, 11% of respondents were moderately to extremely annoyed by traffic noise (54). This analysis suggested that the current wind turbine noise restrictions in Ontario will limit community exposure to wind turbine related noise such that levels of annoyance are unlikely to exceed previously established background levels of noise-related annoyance from other common noise sources.

### Low-frequency noise and infrasound

As reviewed by Knopper and Ollson (9), a number of sources have proposed that the self-reported health effects of some people living near wind turbines may be due to LFN and infrasound [e.g., (20, 39, 55)]. However, infrasound and LFN are not unique to wind turbines; natural sources of infrasound include meteors, volcanic eruptions, ocean waves, wind, and any effect that leads to slow oscillations of the air (11). Measured LFN and infrasound levels from wind turbines have been shown to comply with available standards and criteria published by numerous government agencies including the UK Department for Environment, Food, and Rural Affairs; the American National Standards Institute; and the Japan Ministry of Environment (22). Therefore, Knopper and Ollson (9) concluded that the hypothesis that infrasound is a causative agent in health effects does not appear to be supported. With some exceptions, more recent studies (summarized below) generally support this hypothesis.

Møller and Pedersen (37): Møller and Pedersen conducted a LFN study from four large turbines (>2 MW) and 44 other small and large turbines that were aggregated (7 > 2 and 37 < 2 MW). Low-frequency sound (LFS) insulation was measured for 10 rooms under normal living conditions in houses exposed to LFN. They concluded that the spectrum of wind turbine noise moves down in frequency with increasing turbine size. They also suggested that the low-frequency part of the noise spectrum plays an important role in the noise at neighboring properties. They hypothesized that if the noise from the investigated large turbines had an outdoor level of 44 dB(A) (the maximum of the Danish regulation for wind turbines) there was a risk that a substantial proportion of the residents would be annoyed by LFN, even indoors. However, the authors’ work did not include a survey of annoyance surrounding the turbines and did not provide any data to support this hypothesis. In terms of infrasound (sound below 20 Hz), they concluded that the levels were relatively low when human sensitivity to these frequencies was accounted for. Even in close proximity to turbines, the infrasonic sound pressure level was below the normal hearing threshold. Overall, this study suggested that LFN could be an important component of the overall noise levels from wind turbines. However, it did not provide a link between modeled or measured values and potential health effects of nearby residents. Rather, it hypothesized that at 44 dB(A), at least a portion of the annoyance could be attributed to LFN levels.

Bolin et al. (38): Bolin et al. (38) conducted a literature review over a 6-month period ending April 2011 into the potential health effects related to infrasound and LFN exposure surrounding wind turbines. They conducted the search using PubMed, PsycInfo, and Science Citation Index. In addition, they conducted gray literature searches and personally contacted researchers and noise consultants working with wind turbine noise. They concluded that the dominant source of wind turbine generated LFN was from incoming turbulence interacting with the blades. They found no evidence in the literature that infrasound in the 1–20 Hz range contributed to perceived annoyance or other health effects. They also opined that LFN from modern wind turbines could be audible at typical levels in residential settings, but did not exceed levels from other common noise sources, such as road traffic noise.

The authors concluded that empirical support was lacking for claims that LFN and infrasound cause serious health affects in the form of “vibroacoustic disease (VAD),” “wind turbine syndrome,” or harmful effects on the inner ear. This conclusion was similar to that provided in the Massachusetts Department of Environmental Protection (MassDEP) and Massachusetts Department of Public Health (MDPH) expert panel review released in January 2012.

Rand et al. (39) and Ambrose et al. (40): in the fall of 2011, Rand et al. published their findings on noise measurements taken around a residential home online in the Bulletin of Science, Technology and Society (BSTS) (39). In 2012, a similar article appeared in BSTS, but with Ambrose as first author. After learning about reported noise and health issues from some residents living near three wind turbines (Vestas, Model V82, 1.65 MW each) in Falmouth, MA, USA, Ambrose et al. conducted a study to investigate the role of infrasound and LFS in these complaints. What led Ambrose et al. to focus on infrasound and LFS was the home owner’s complaints about discomfort and a number of symptoms (i.e., headaches, ear pressure, dizziness, nausea, apprehension, confusion, mental fatigue, inability to concentrate, and lethargy). These observations were reported to be associated with being indoors when the wind turbines were operating during moderate to strong winds. Ambrose et al. state: *“Typically, indoors the A-weighted sound level is lower than outdoors when human activity is at a minimum. This strongly suggested that the A-weighted sound level might not correlate very well [sic] the wind turbine complaints. This may be indicative of another cause such as low- or very-low-frequency energy being involved.”*

The authors made acoustic measurements and viewed the data with dBL (unweighted) and dB(A), (C), and (G) filtering between April 17 and 19, 2011, at four locations [260 ft (~87 m), 830 ft (~277 m), 1,340 ft (~450 m), and 1,700 ft (~570 m)] between one turbine and one residence. The relationship between sound [dB(A), (G), and (L)] and health effects was based on measurements at 1,700 ft. Ambrose et al. reported that within 20 min, both authors had difficulties performing ordinary tasks and within 1 h both were “*debilitated and had to work much harder mentally.”* They also claimed that as time went on their symptoms became more severe.

The authors reported being affected when wind speeds were greater than 10 m/s at the hub height of the turbines and when measured sound levels were in the 18–24 dB(A) range inside [51–64 dB(G); 62–74 dB(L)] and 32–46 dB(A) outside [49–65 dB(G); 57–69 dB(L)]. They reported that they felt effects inside and outside but preferred being outside. They noted that it took a week to recover but one researcher had recurring symptoms (of nausea and vertigo) for over 7 weeks. There are a number of uncertainties in the Ambrose et al. white paper and the BSTS articles, which diminished the strength of their conclusions. This was the first written account we are aware of that suggested acute health effects from exposure to sound from wind turbines. The recent MassDEP and MDPH (56) report provided this comment regarding the Ambrose et al. study: *“Importantly, while there is an amplification at these lower frequencies, the indoor levels (unweighted) are still far lower than any levels that have ever been shown to cause a physical response (including the activation of the OHC) in humans.”*

Further, studies where biological effects observed following infrasound exposure were conducted at sound pressure levels much greater than measured by Ambrose et al. [e.g., (11); 145 and 165 dB; (57): 130 dB] and much greater than what is produced by wind turbines. There are over 100,000 wind turbines in operation globally. Indeed, the idea of overt acute debilitating effects (even lasting several weeks after removal from exposure) appears to be unique to these authors.

Turnbull et al. (41): Turnbull et al. developed an underground technique to measure infrasound and applied this process at two Australian wind farms as well as in the vicinities of a beach, a coastal cliff, the city of Adelaide, and a power station. The measured levels were compared against one another and against the infrasound audibility threshold of 85 dB(G). The authors reported that the measured level of infrasound within the wind farms was well below the audibility threshold and was similar to that of urban and coastal environments and near other engineered noise sources. Indeed, the level of infrasound from wind farms at 360 and 85 m [61 and 72 dB(G), respectively] was comparable to that observed at a distance of 25 m from ocean waves [75 dB(G)].

Crichton et al. (7): this study examined the possibility that expectations of negative health effects from exposure to infrasound promote symptom reporting. A sham controlled, double-blind provocation study was conducted in which participants were exposed to 10 min of infrasound and 10 min of sham infrasound. A total of 54 participants (34 women, 20 men) were randomized into high- or low-expectancy groups and presented with audiovisual information (including internet material) designed to invoke either high or low expectations that exposure to infrasound causes specific symptoms (e.g., headache, ear pressure, itchy skin, sinus pressure, dizziness, vibrations within the body). Notably, participants in the high-expectancy group reported significant increases in the number and intensity of symptoms experienced during exposure to both infrasound and sham infrasound. Conversely, there were no symptomatic changes in the low-expectancy group.

Based on their findings, Crichton et al. (7) concluded: *“Healthy volunteers, when given information about the expected physiological effect of infrasound, reported symptoms that aligned with that information, during exposure to both infrasound and sham infrasound. Symptom expectations were created by viewing information readily available on the Internet, indicating the potential for symptom expectations to be created outside of the laboratory, in real world settings. Results suggest psychological expectations could explain the link between wind turbine exposure and health complaints.”* These results were consistent with the findings of other researchers, who have observed increased concern about the health risks associated with exposure to certain environmental hazards can lead to elevated symptom reporting, even when no objective health risk is presented (58, 59).

Crichton et al. (8): building on their previous publication that negative expectations established by the media and internet can significantly increase health-related complaints by exposed individuals (8), the authors investigated how positive expectations can produce a reduction in symptoms. Sixty participants were exposed to audible wind farm sound [43 dB(A)] and infrasound [9 Hz, 50.4 dBL (unweighted)] previously recorded 1 km from a wind farm, in two, 7 min session. Following baseline measurements, expectations were developed by watching videos that either promoted the negative health effects or the potentially therapeutic health effects of exposure to infrasound. Expectations were found to significantly alter symptom reporting: participants who were primed with negative expectations became more symptomatic over time, suggesting that their experiences during the first exposure session reinforced expectations and led to heightened symptomatic experiences in subsequent sessions. Upwards of 77% of participants in the negative expectation group reported a worsening of symptoms. In contrast, 90% of participants in the positive expectation group reported improvements in physical symptoms after the listening session. This was the first study to show that a placebo response could be brought on by positive pre-exposure expectations and influence participants exposed to wind farm noise. The authors concluded that negative expectations created by the media could account for the increase in negative health effects reported by individuals exposed to wind farm noise. Overall, this investigation provided further evidence that physiological outcomes can be influenced by established expectations.

### Electromagnetic Fields

Concerns about the ever-present nature of EMF (also called electric and magnetic fields) and possible health effects have been raised by some in the global community for a number of years. However, the science around EMF and possible health concerns has been extensively researched, with tens of thousands of scientific studies published on the issue. Government and medical agencies including Health Canada (60), the World Health Organization (61), the International Commission on Non-Ionizing Radiation Protection (62), the International Agency for Research on Cancer (63), and the US National Institute of Health (NIH) and National Institute of Environmental Health Sciences (64) have all thoroughly reviewed the available information. While individual opinions on the issue vary, the weight of scientific evidence does not support a causal link between EMF and health issues at levels typically encountered by people.

Short-term exposure to EMF at high levels is known to cause nerve and muscle stimulation in the central nervous system. Based on this information, the ICNIRP, a group recognized by the WHO as the international independent advisory body for non-ionizing radiation protection, established an acute exposure guideline of 2,000 mG for the general public, based on power frequency EMF of 50–400 Hz (62). With respect to long-term exposure to low levels of EMF, it needs to be acknowledged that the IARC and WHO have categorized EMF as a Class 2B possible human carcinogen, based on a weak association of childhood leukemia and magnetic field strength above 3–4 mG (63). This means there is limited evidence of carcinogenicity in humans and inadequate evidence of carcinogenicity in experimental animals. These human studies are weakened by various methodological problems that the WHO has identified as a combination of selection bias, some degree of confounding and chance (65). There are also no globally accepted mechanisms that would suggest that low-level exposures are involved in cancer development and animal studies have been largely negative (65). Thus, the WHO has stated that, based on approximately 25,000 articles published over the past 30 years, the evidence linking childhood leukemia to EMF exposure is not strong enough to be considered causal (61). Concerns have also been raised by some about a relationship between EMF and a range of various health concerns, including cancers in adults, depression, suicide, and reproductive dysfunction, among several others. The WHO (65) has stated: *“*…*scientific evidence supporting an association between ELF [extremely low frequency] magnetic field exposure and all of these health effects is much weaker than for childhood leukaemia.”*

Recently, worries about exposure to EMF from wind turbines, and associated electrical transmission, has been raised at public meetings and legal proceedings. These fears have not been based on any actual measurements of EMF exposure surrounding existing projects but appear to follow from concerns raised from internet sources and misunderstanding of the science. There has been limited research conducted on wind turbine emissions of EMF, either from the turbines themselves, or from the power lines required for distribution of the generated electricity. However, based on the weight of evidence it is not expected that EMF from wind turbines is likely to be a causative agent for negative health effects in the community. Only three papers were retrieved in the preparation of this review that examined this issue specifically.

Havas and Colling (42): the paper indicated that there were some people who lived around wind turbines that complained of difficulty sleeping, fatigue, depression, irritability, aggressiveness, cognitive dysfunction, chest pain/pressure, headaches, joint pain, skin irritations, nausea, dizziness, tinnitus, and stress. The authors suggested that these symptoms could be caused by electromagnetic waves in the form of poor power quality (dirty electricity) and ground current resulting in health effects in those that are electrically hypersensitive. They indicated that individuals reacted differently to both sound and electromagnetic waves and this could explain why not everyone experienced the same health effects living near turbines. Ground current or stray voltage was also purported to be a potential cause of health effects surrounding wind turbines. However, this paper was hypothetical and speculative in nature and no data were presented to support the author’s opinions. Presently, there are no quantitative data in the scientific literature to support the claims made in Havas and Colling (42).

Israel et al. (43): these authors conducted EMF, sound, and vibration measurements surrounding one of the largest wind energy parks in Bulgaria, located along the Black Sea. The purpose of the study was to determine if levels of wind turbine emissions were within Bulgarian and European limits for workers and the general population. In addition, they sought to determine if their previously established 500 m setback zone around the wind park was adequate. The wind park consisted of 55 Vestas V90 3 MW towers. The measurements took place over a 72-h period when temperatures were between 0 and 5.5°C. Actual distances to the receptor locations were not reported, although it is suspected that they would be in the vicinity of 500 m from the closest turbines.

The EMF levels measured within 2–3 m of the wind turbines were between 0.133 and 0.225 mG. These values are comparable to or lower than magnetic field measurements that have been reported in the proximity of typical household electrical devices (66). It should be noted that the values observed by Israel et al. were approximately four orders of magnitude lower than the ICNIRP (62) guideline of 2,000 mG for the general public for acute exposure. Based on these findings, Israel et al. concluded that the EMF levels from wind turbines were at such low level as to be insignificant compared to values found in residential areas and homes. The findings reported by Israel et al. of actual measurements of EMF surrounding wind turbines were contrary to the hypothesis presented by Havas and Colling (42).

The noise measurements performed by Israel et al. met the requirements of Bulgarian legislation for day [55 dB(A)], evening [50 dB(A)], and night [45 dB(A)] and it was concluded that the wind turbines contributed only 1–3 dB(A) above existing background levels. Vibration measurements surrounding the turbines had values close to zero, which indicated that this was not a contributing emission factor of exposure for people living around wind turbines. Overall, the authors concluded:*“*…*the studied wind power park complies with the requirements of the national and European legislation for human protection from physical factors–electric and magnetic fields up to 1 kHz, noise, vibration, and do not create risk for both workers in the area of the park and the general population living in the nearest villages.”*

McCallum et al. (44): this study was carried out at the Kingsbridge 1 Wind Farm located near Goderich, ON, Canada. Magnetic field measurements (milligauss) were collected in the proximity of 15 Vestas 1.8 MW wind turbines, two substations, various buried and overhead collector and transmission lines, and nearby homes. Data were collected during three operational scenarios to characterize potential EMF exposure: “high wind” (generating power), “low wind” (drawing power from the grid, but not generating power), and “shut off” (neither drawing, nor generating power).

Background levels of EMF (0.2–0.3 mG) were established by measuring magnetic fields around the wind turbines under the “shut off” scenario. Magnetic field levels detected at the base of the turbines under both the “high wind” and “low wind” conditions were low (mean = 0.9 mG; *n* = 11) and rapidly diminished with distance, becoming indistinguishable from background within 2 m of the base. Magnetic fields measured 1 m above buried collector lines were also within background (≤0.3 mG). Beneath overhead 27.5 and 500 kV transmission lines, magnetic field levels of up to 16.5 and 46 mG, respectively, were recorded. These levels also diminished rapidly with distance. None of these sources appeared to influence magnetic field levels at nearby homes located as close as just over 500 m from turbines, where measurements immediately outside of the homes were ≤0.4 mG. The results suggested that there was nothing unique to wind farms with respect to EMF exposure; in fact, magnetic field levels in the vicinity of wind turbines were lower than those produced by many common household electrical devices (e.g., refrigerator, dishwasher, microwave, hairdryer) and were well below any existing regulatory guidelines with respect to human health.

### Shadow Flicker

The main health concern associated with shadow flicker is the risk of seizures in those people with photosensitive epilepsy. As reviewed by Knopper and Ollson (9), Harding et al. (14) and Smedley et al. (19) have published the seminal studies dealing with this concern. Both authors investigated the relationship between photo-induced seizures (i.e., photosensitive epilepsy) and wind turbine blade flicker (also known as shadow flicker). Both studies suggested that flicker from turbines that interrupt or reflect sunlight at frequencies >3 Hz pose a potential risk of inducing photosensitive seizures in 1.7 people per 100,000 of the photosensitive population. For turbines with three blades, this translates to a maximum speed of rotation of 60 rpm. Modern turbines commonly spin at rates well below this threshold. For example, the following spin rates for four different models of wind turbines have been obtained from the turbine specification sheets:

- Siemens SWT-2.3: 6–16 rpm
- REpower MM92: 7.8–15.0 rpm
- GE 1.6–100: 9.75–16.2 rpm
- Vestas V112-3.0: 6.2–17.1 rpm

In 2011, the Department of Energy and Climate Change (67) released a consultant’s report entitled “Update of UK Shadow Flicker Evidence Base.” The report concluded that: *“On health effects and nuisance of the shadow flicker effect, it is considered that the frequency of the flickering caused by the wind turbine rotation is such that it should not cause a significant risk to health.”* Furthermore, the expert panel convened by MassDEP and MDPH (56) concluded that the scientific evidence suggests that shadow flicker does not pose a risk of inducing seizures in people with photosensitive epilepsy.

Germany is one of the only countries to implement formal shadow flicker guidelines, which are part of the *Federal Emission Control Act* (68). These guidelines allow:

- maximum 30 h per year of astronomical maximum shadow (worst case);
- maximum 30 min worst day of astronomical maximum shadow (worst case); and
- maximum 8 h per year actual.

Although shadow flicker from wind turbines is unlikely to lead to a risk of photo-induced epilepsy, there has been little if any research conducted on how it could heighten the annoyance factor of those living in proximity to turbines. It may however be included in the notion of visual cues.

### Review Articles, Editorials, and Social Commentaries

In addition to the articles reviewed above that reported the results of surveys and experiments designed to specifically investigate potential environmental stressors that have been associated with wind turbines (i.e., overall noise, LFN and infrasound, EMF, and shadow flicker), a number of published and peer-reviewed articles were identified that present reviews of the available data, opinion pieces, and/or social commentaries. These articles are reviewed in detail below.

Bulletin of Science, Technology and Society: Special Edition 2011, 31(4): in August 2011, authors of a number of popular literature studies published their findings as a series of nine articles in a special edition of the Bulletin of Science, Technology and Society (BSTS) devoted entirely to wind farms and potential health effects^1^. Many of the articles in the special edition were written as opinion pieces or social commentaries and did not provide detailed methodologies used to test hypotheses as is expected in the publication of scientific research articles. Based on a critical review of each of the articles (69), it is our opinion that the series suffers numerous flaws from a scientific, technological, and social basis. Many of the claims used as evidence of a relationship between health effects and wind turbines were unsubstantiated [e.g., Phillips (70) is entirely unsupported and contains alarmist extrapolations], without proper references [e.g., (70, 71)] and based on anecdotal or unconfirmed reports [e.g., (55, 70, 72, 73)], fallacious comparisons [e.g., (74)], and reaching arguments lacking a logical process [e.g., (70, 73, 75, 76)]. Further, much information given as fact was contrary to that published in the scientific literature; indeed, many authors appeared to selectively reference articles and information in a way that would benefit their own arguments [e.g., (55, 71)]. The results of this BSTS special issue failed to provide valid, defensible scientific and social arguments to suggest that wind turbines, regardless of siting considerations, cause harm to human health.

Hanning and Evans (45) and Chapman (46): in 2012, Hanning and Evans had an editorial published in the British Medical Journal (BMJ), the purpose of which was to opine on the relationship between wind turbines noise and health effects. By citing a short list of articles (12), half of which are from the non-indexed journal BSTS or from conference proceedings (3 and 3, respectively, out of 12), Hanning and Evans suggested that: *“A large body of evidence now exists to suggest that wind turbines disturb sleep and impair health at distances and external noise levels that are permitted in most jurisdictions.”* and *“Robust independent research into the health effects of existing wind farms is long overdue, as is an independent review of existing evidence and guidance on acceptable noise levels.”*

Shortly after publication, this editorial was rebuffed by Chapman (46), in another editorial placed in the BMJ. Chapman pointed out that there are a number of independent reviews of the literature around wind turbines and human health (Chapman points to 17 such papers not referenced by Hanning and Evans). Chapman opined that: *“These reviews strongly state that the evidence that wind turbines themselves cause problems is poor. They conclude that: Small minorities of exposed people claim to be adversely affected by turbines; Negative attitudes to turbines are more predictive of reported adverse health effects and annoyance than are objective measures of exposure; Deriving income from hosting wind turbines may have a “protective effect” against annoyance and health symptoms.”* Further debate about the original editorial is available online to view (and comment on) through the BMJ web site^2^.

Farboud et al. (47): this review article looked at the effects of LFN and infrasound and questioned the existence of “wind turbine syndrome.” The authors conducted a literature search for articles published within the last 10 years, using the PubMed database and the Google Scholar search engine. Their search terms included “wind turbine,” “infrasound,” or “LFN” and search results were limited to the English language, human trials, and either randomized control trials, meta-analyses, editorial letters, clinical trials, case reports, comments, or journal articles. A number of articles dealing with “wind turbine,” “infrasound,” or “LFN,” and available in PubMed and Google Scholar, appear to have been missed by Farboud et al. [e.g., (9, 22, 38)]. The review included discussions on topics such as wind turbine noise measurements and regulations, wind turbine syndrome, and the effects of LFN and infrasound.

The authors discussed the use of A-weighting in noise measurements from wind turbines stating: *“The A-filter de-emphasizes all auditory energy with frequencies of less than 500 Hz, and completely ignores all auditory energy of less than 20 Hz, in an effort to estimate the noise thought to be actually processed by the ear. Hence, much of the noise produced by a wind turbine is effectively ignored.”* The authors later described the results and implications of studies looking at the effects of infrasound in the ear, and noted that infrasound and LFN are currently not recognized as disease agents. Referencing a study by Salt and Hullar (20), the authors noted that the inner hair cells of the cochlea, which is the main hearing pathway in mammals, are not sensitive to infrasound. Conversely, the outer hair cells of the cochlea are more sensitive to LFN and infrasound and can be stimulated at levels below the auditory threshold. Nevertheless, the authors conceded that: *“*…*low-frequency noise may well influence inner ear physiology. However, whether this actually alters function or causes symptoms is unknown.”*

It should be noted that, as discussed in the “Low-Frequency Noise and Infrasound” section of this review, there were a number of studies that specifically addressed the concerns of LFN and infrasound from wind turbines that suggested that these were unlikely to be causative agents in health effects of those living near wind turbines [e.g., (7, 11, 22, 37, 38)]. Unfortunately, none of these studies were included as part of the Farboud et al. review.

Regarding the existence of “Wind Turbine Syndrome,” Farboud et al. stated that: *“There is an abundance of information available on the internet describing the possibility of wind turbine syndrome. However, the majority of this information is based on purely anecdotal evidence.”* The authors briefly discussed the various symptoms that have been self-reported by individuals and attributed to noise from wind turbines. They also pointed out that “Wind Turbine Syndrome” was not a clinically recognized diagnosis, remained unproven, and was not generally accepted within the scientific and medical community. They also mentioned that some researchers maintained that the effects of “Wind Turbine Syndrome” were just examples of the well-known stress effects of exposure to noise, as displayed by a small proportion of the population.

Farboud et al. concluded their review by suggesting that the evidence available was incomplete and until the physiological effects of LFN and infrasound were fully understood, it was not possible to conclusively state that wind turbines were not causing any of the reported effects. However, it was not clear how this conclusion might have been altered had they considered the additional available information regarding LFN and infrasound from wind turbines described elsewhere in this review [i.e., (7, 11, 22, 37, 38)].

McCubbin and Sovacool (48): McCubbin and Sovacool (48) presented a comparison of the health and environmental benefits of wind power in contrast to natural gas. The authors selected two locations: the 580 MW wind farm at Altamont Pass in California and the 22 MW wind farm in Sawtooth, ID, USA. The paper considered the environmental and economic benefits associated with each wind farm. Human health benefits were calculated based on a reduction in ambient PM_2.5_ levels using well-established health impact and valuation functions from the US EPA. Additionally, benefits to the health and well-being of wildlife and avian species were quantified.

With regard to the human health impacts, the potential cost savings were associated with effects such as premature mortality, hospital admissions, emergency rooms visits, asthma attacks, and respiratory symptoms. The details of the quantification methods and equations used to calculate the benefits to externalities such as human health, wildlife, and the natural environment were not provided herein but are available in the published manuscript.

McCubbin and Sovacool determined that from 2012 to 2031 the wind turbines at Altamont Pass will avoid anywhere from $560 million to $4.38 billion in human health and climate-related externalities, and the Sawtooth wind farm will avoid from $18 million to $24 million. The authors noted that there were uncertainties associated with their quantification methods and final cost estimates; however, they claimed that the values were likely underestimated based on numerous factors that were not considered (e.g., other pollutants). They concluded that: *“Despite the uncertainties, the evidence gathered here strongly suggests that natural gas had substantial external costs that should be included in an evaluation comparing wind energy to combined cycle natural gas-fired power plants. The overall costs of electricity generated by natural gas are greater than those from wind energy when environmental and human health externalities are quantified. It remains likely that over time the relative difference will widen, making the use of wind energy even more favorable.”*

Roberts and Roberts (49): the authors conducted a summary of the peer-reviewed literature on the research that examined the relationship between human health effects and exposure to LFS and sound generated from the operation of wind turbines. The PubMed database (maintained by the US National Library of Medicine) was relied upon for retrieving the peer-reviewed literature used in this review. A number of search terms were used including: “infrasound and health effects”; “LFN and health effects”; “LFS and health effects”; “wind power and noise”; and “wind turbines AND noise.” In total, 156 articles were identified with 28 articles addressing health effects and LFS related to wind turbines. Based on the collective results of the studies reviewed, Roberts and Roberts (49) found that: *“At present, a specific health condition or collection of symptoms has not been documented in the peer-reviewed, published literature that has been classified as a ‘disease’ caused by exposure to sound levels and frequencies generated by the operations of wind turbines. It can be theorized that reported health effects are a manifestation of the annoyance that individuals experience as a result of the presence of wind turbines in their communities.”*

Chapman and St. George (50): in 2007, Alves-Pereira and Castelo Branco issued a press-release suggesting that their research demonstrated that living in proximity to wind turbines had led to the development of VAD in nearby home-dwellers (9). Alves-Pereira and Castelo Branco appear to be the primary researchers who have circulated VAD as a hypothesis for adverse health effects and wind turbines and to our knowledge this work has never appeared in a peer-reviewed article. In this paper, Chapman and St. George investigated the extent to which VAD and its alleged association with wind turbine exposure had received scientific attention, the quality of that association, and how the alleged association gained support by wind farms opponent.

Based on a structured scientific database and Google search strategy, the authors showed that *“VAD has received virtually no scientific recognition beyond the group who coined and promoted the concept. There is no evidence of even rudimentary quality that vibroacoustic disease is associated with or caused by wind turbines.”* They went on to state that an implication of this “factoid” – defined as questionable or spurious statements – may have been contributing to nocebo effects among those living near turbines. That is the spread of negative, often emotive information would be followed by increases in complaints and that without such suggestions being spread, complaints would be less. These results highlighted the role that perception plays in the human health wind turbine debate and underscored the role of proper risk communication in communities.

Jeffery et al. (51, 52): the overall goal of these commentary pieces was to provide information to physicians regarding the possible health effects of exposure to noise produced by wind turbines and how these may manifest in patients. In the 2013 article, information about the *Green Energy Act* was presented in such a way that implied that the overall goal of the Act was to remove protective noise regulations and allow wind turbines to be placed “in close proximity to family homes.” The authors suggested that there has been a concerted effort to minimize the potential health risks while convincing the general public and physicians that wind turbines are beneficial. No evidence was given to support these claims. Case reports and publications that reported adverse effects following wind turbines noise exposure were briefly discussed; however, only the negative health effects were highlighted. Older literature and a number of non-peer-reviewed articles and media reports were used to support the author’s opinions. The 2014 paper is very similar to that published in 2013. The authors provided a very one-sided opinion in their review of the issue of wind turbines and adverse health effects. They have missed a number of key and pertinent articles that have been published on the issue. Overall the authors did not provide adequate data or support for their arguments, in both papers, nor did they provide accurate information regarding the weight of scientific data on the issue.

## Weight of Evidence Conclusions

There are roughly 60 studies that have been conducted worldwide on the issue of wind turbines and human health. In terms of effects being related to wind turbine operational effects and wind turbine noise, there are fewer than 20 articles. The vast majority has been published in one journal (BSTS) and many of these authors sit on advisory board of the Society for Wind Vigilance, an advocacy group in the province of Ontario. However, with respect to effects being more likely attributable to a number of subjective variables (when turbines are sited correctly), there are closer to 45 articles. These articles are published by a variety of different authors with wide and diverse affiliations. Indeed, conclusions stemming from these articles are supported by studies where audible and inaudible noise has been quantified from operational wind turbines.

Based on the findings and scientific merit of the research conducted to date, it is our opinion that the weight of evidence suggests that when sited properly, wind turbines are not related to adverse health effects. This claim is supported (and made) by findings from a number of government health and medical agencies and legal decisions [e.g., (56, 77–80)]. Collectively, the evidence has shown that while noise from wind turbines is not loud enough to cause hearing impairment and is not causally related to adverse effects, wind turbine noise can be a source of annoyance for some people and that annoyance may be associated with certain reported health effects (e.g., sleep disturbance), especially at sound pressure levels >40 dB(A).

The reported correlation between wind turbine noise and annoyance is not unexpected as noise-related annoyance [described by Berglund and Lindvall (81) as a *“feeling of displeasure evoked by a noise”*] has been extensively linked to a variety of common noise sources such as rail, road, and air traffic (81–83). Noise-related annoyance from these more common sources is prevalent in many communities. For instance, results of national surveys in Canada and the U.K. by Michaud et al. (54) and Grimwood et al. (84), respectively, suggested that annoyance from noise (predominantly traffic noise) may impact approximately 8% of the general population. Even in small communities in Canada (i.e., <5000 residents) where traffic is relatively light compared to urban centers, Michaud et al. (54) reported that 11% of respondents were moderately to extremely annoyed by traffic noise.

Although annoyance is considered to be the least severe potential impact of community noise exposure (83, 85), it has been hypothesized that sufficiently high levels of annoyance could lead to negative emotional responses (e.g., anger, disappointment, depression, or anxiety) and psychosocial symptoms (e.g., tiredness, stomach discomfort, and stress) (83, 86–90). However, it is important to note that noise annoyance is known to be strongly affected by attitudinal factors such as fear of harm connected with the source and personal evaluation of the source (91–93) as well as expectations of residents (92). For wind turbines, this has been reflected in studies that have shown that subjective variables like evaluations of visual impact (e.g., beautiful vs. ugly), attitude to wind turbines (benign vs. intruders), and personality traits are more strongly related to annoyance and health effects than noise itself [e.g., (4, 5, 16, 17, 31)]. Thus, it is likely that the adverse effects exhibited by some people who live near wind turbines are a response to stress and annoyance, which are driven by multiple environmental and personal factors, and are not specifically caused by any unique characteristic of wind turbines. This hypothesis is also supported by the observation that people who economically benefit from wind turbines have significantly decreased levels of annoyance compared to individuals that received no economic benefit, despite exposure to similar, if not higher, sound levels (17).

There is also a growing body of research that suggests that nocebo effects may play a role in a number of self-reported health impacts related to the presence of wind turbines. Negative attitudes and worries of individuals about perceived environmental risks have been shown to be associated with adverse health-related symptoms such as headache, nausea, dizziness, agitation, and depression, even in the absence of an identifiable cause (94–96). Psychogenic factors, such as the circulation of negative information and priming of expectations have been shown to impact self-assessments following exposure to wind turbine noise (6–8). It is therefore important to consider the role of mass media in influencing public attitudes about wind turbines and how this may alter responses and perceived health impacts of wind turbines in the community. For example, Deignan et al. (97) recently demonstrated that newspaper coverage of the potential health effects of wind turbines in Ontario has tended to emphasize “fright factors” about wind turbines. Specifically, Deignan et al. (97) reported that 94% of articles provided *“negative, loaded or fear-evoking”* descriptions of *“health-related signs, symptoms or adverse effects of wind turbine exposure”* and 58% of articles suggested that the effects of wind turbines on human health were *“poorly understood by science.”* It is possible that this type of coverage may have a significant impact on attitudinal factors, such as fear of the noise source, that are known to increase noise annoyance (91–93).

Stress/annoyance is not unique to living in proximity to wind turbines. The American Psychological Association (98) published a report stating that the majority of Americans are living with moderate (4 to 7 on a scale of 1 to 10) or high (8 to 10 on a scale of 1 to 10) levels of stress. APA identified money, work, and the economy as the most often cited sources of stress in Americans followed by family responsibilities, relationships, job stability, housing costs, health concerns, health problems, and safety. Stress from these and other sources can lead to a number of adverse health effects that are commonplace in society. The Mayo Clinic (99) identifies irritability, anger, anxiety, sadness/guilt, change in sleep, fatigue, difficulty concentrating or making decisions, loss of interest/enjoyment, nausea, headache, and tinnitus as common symptoms of stress. Interestingly, these symptoms are nearly identical to those suggested by McMurtry (55) as criteria for a *“diagnosis of adverse health effects in the environs of industrial wind turbines.”*

Based on the available evidence, we suggest the following best practices for wind turbine development in the context of human health. However, it should be noted that subjective variables (e.g., attitudes and expectations) are strongly linked to annoyance and have the potential to facilitate other health complaints via the nocebo effect. Therefore, it is possible that a segment of the population may remain annoyed (or report other health impacts) even when noise limits are enforced.

1. Setbacks should be sound-based rather than distance-based alone.
2. Preference should be given to sound emissions of ≤40 dB(A) for non-participating receptors, measured outside, at a dwelling, and not including ambient noise. This value is the same as the WHO (Europe) night noise guideline (100) and has been demonstrated to result in levels of wind turbine community annoyance similar to, or lower than, known background levels of noise-related annoyance from other common noise sources.
3. Post construction monitoring should be common place to ensure modeled sound levels are within required noise limits.
4. If sound emissions from wind projects is in the 40–45 dB(A) range for non-participating receptors, we suggest community consultation and community support.
5. Setbacks that permit sound levels >45 dB(A) (wind turbine noise only; not including ambient noise) for non-participating receptors directly outside a dwelling are not supported due to possible direct effects from audibility and possible levels of annoyance above background.
6. When ambient noise is taken into account, wind turbine noise can be >45 dB(A), but a combined wind turbine-ambient noise should not exceed >55 dB(A) for non-participating and participating receptors. Our suggested upper limit is based on WHO (100) conclusions that noise above 55 dB(A) is “*considered increasingly dangerous for public health*,” is when “*adverse health effects occur frequently, a sizeable proportion of the population is highly annoyed and sleep-disturbed*” and “*cardiovascular effects become the major public health concern, which are likely to be less dependent on the nature of the noise*.”

Over the past 20 years, there has been substantial proliferation in the use of wind power, with a global increase of over 50-fold from 1996 to 2013 (1). Such an increase of investment in renewable energy is a critical step in reducing human dependency on fossil fuel resources. Wind-based energy represents a clean resource that does not produce any known chemical emissions or harmful wastes. As highlighted in a recent editorial in the British Medical Journal, reducing air pollution can provide significant health benefits, including reducing asthma, chronic obstructive pulmonary disease, cancer, and heart disease, which in turn could provide significant savings for health care systems (101). By following our proposed health-based best practices for wind turbine siting, wind energy developers, the media, members of the public and government agencies can work together to ensure that the full potential of this renewable energy source is met.

## Author Contributions

All authors contributed in varying degrees to writing, editing, and reviewing this manuscript.

## Conflict of Interest Statement

In terms of competing interests (financial and non-financial), the authors work for a consulting firm and have worked with wind power companies. The authors are actively working in the field of wind turbines and human health. Although we make this disclosure, we wish to reiterate that as independent scientific professionals our views and research are not influenced by these contractual obligations. The authors are environmental health scientists, trained and schooled, in the evaluation of potential risks and health effects of people and ecosystems through their exposure to environmental issues such as wind turbines.

## Footnotes

^1^http://bst.sagepub.com/

^2^http://www.bmj.com/content/344/bmj.e1527?tab=responses
